# Understanding the complexities of oral healthcare delivery in correctional settings: a qualitative exploration of barriers, facilitators, and opportunities

**DOI:** 10.1186/s12889-025-24447-9

**Published:** 2025-09-09

**Authors:** Arianna Amaya, Ivan Medina, Farimah Rezaei, Pegah Soleimani, Rebecca Bosworth, Heino Stöver, Babak Moazen

**Affiliations:** 1 Heidelberg Institute of Global Health, Heidelberg University, Bergheimer Str. 20, Zimmer 317, 69115 Heidelberg, Germany; 2 School of Economic and Administrative Sciences, Corporación Universitaria de Asturias, Bogotá, Colombia; 3 Faculty of Medicine, Isfahan University of Medical Sciences, Isfahan, Iran; 4 Faculty of Medicine and Dentistry, University of Alberta, Edmonton, AB Canada; 5 National Drug and Alcohol Research Centre (NDARC), University of New South Wales (UNSW), Sydney, Australia; 6 Justice Health and Forensic Mental Health Network (JHNSW), Sydney, Australia; 7 Faculty of Health, School of Nursing, Queensland University of Technology (QUT), Brisbane, Australia; 8 Faculty of Science, Medicine and Health, School of Nursing, University of Wollongong, Wollongong, Australia; 9 Department of Health and Social Work, Institute of Addiction Research (ISFF), Frankfurt University of Applied Sciences, Frankfurt/Main, Germany

**Keywords:** Oral health, Oral health-related quality of life, Prisons, Public health, Health promotion

## Abstract

**Background:**

People living in prison face exceptionally high prevalence rates of tooth decay, periodontal disease, and poor oral health-related quality of life. Despite its importance, various aspects of oral healthcare in prison settings remain understudied. The present study investigates the barriers and facilitators associated with providing and utilizing oral health services in prison settings, drawing on insights from prison health experts, managerial and custodial staff, healthcare providers, and individuals with lived experience of imprisonment.

**Methods:**

From March to June 2023, a total of fifteen participants participated in semi-structured in-depth interviews. Interviews were conducted until data saturation to identify barriers and facilitators of oral health services in prisons. Potential areas for improvement were also explored. Thematic analysis was used to analyze the data. Themes and sub-themes were derived from the dataset and converted into preliminary codes which aligned with research objectives.

**Results:**

The first topic, barriers, included two themes: organizational barriers related to the provision of services, and individual barriers related to the utilization of services. The second topic, facilitating factors, included six themes: funding, community partnership, substance use treatment, communicating with policy makers, transportation, and education.

**Conclusion:**

Ensuring the oral health of incarcerated individuals is a fundamental aspect of their right to health and a crucial factor in their successful reintegration into society. Formerly incarcerated people are often hard to reach in the community, making the period of confinement a unique opportunity to deliver quality oral healthcare. Given that most incarcerated individuals will eventually return to their communities, providing comprehensive healthcare, including oral health interventions, represents a valuable public health investment.

**Supplementary information:**

The online version contains supplementary material available at 10.1186/s12889-025-24447-9.

## Introduction

According to the World Health Organization (WHO) “Oral health is the state of the mouth, teeth and orofacial structures that enables individuals to perform essential functions such as eating, breathing and speaking, and encompasses psychosocial dimensions such as self-confidence, well-being and the ability to socialize and work without pain, discomfort and embarrassment.” [1] Oral disorders, including dental caries, periodontitis, and tooth loss, are among the most common chronic health conditions affecting nearly half of the global population [2]. Oral health is not solely determined by individual dental hygiene habits, it is also influenced by population, social, psychological, and systemic factors [3]. Despite being a key component of quality of life [4], many people worldwide, especially priority populations, lack access to adequate oral health services [5].

People living in prisons (PLP) are a high-priority group that experience significant challenges with poor oral health outcomes [6]. Currently, over 11 million people are held in prison settings worldwide [7], many of whom experience poor general and oral health conditions. According to WHO, people in contact with criminal justice systems experience high levels of tooth decay, with prevalence rates ranging up to 67% [8]. They also face a high prevalence of periodontal disease, oral cancers, and poor oral health-related quality of life, all of which impact their daily functioning [8]. Oral health among the prison population is more than just an aesthetic concern, as poor oral health can hinder the reintegration of PLP into society upon release [6]. This aligns with evidence from the literature showing a higher prevalence of missing and decayed teeth among unemployed individuals compared to their employed counterparts [9–11].

Oral diseases are associated with various modifiable risk factors, including excessive sugar intake, substance use, poor oral hygiene, and the underlying social and economic determinants which magnify these risk factors [12]. In prisons, additional risk factors for poor oral health include high levels of illicit drug use, widespread interpersonal violence, deprioritization of personal hygiene, and the limited availability, accessibility, affordability, and quality of oral health prevention and treatment interventions [6]. Substance use, a key contributor to poor oral health, is notably higher among prison populations than in the general population. For instance, in Europe, the lifetime prevalence of illicit drug use prior to incarceration has been estimated to reach up to 90% among PLP [13].

Health professionals such as dentists, nurses, doctors and allied health, as well as non-health professionals, such as prison officers and food service workers, are essential in promoting oral health within custodial facilities. However, the scope and impact of their roles in relation to oral health promotion are not well understood. The role of prison officers has been underexplored and, in some cases, ignored, in implementation of policy to create health promoting prisons [14]. In relation to oral health, the stark deficit of oral health professionals in custodial settings has been documented [8]. This uneven distribution of oral health professionals calls for an interprofessional approach to oral health promotion, where a range of non-dental professionals can work collaboratively to address the various factors that impact oral health outcomes among PLP [15]. From a public health perspective, flexible, innovative workforce models must be explored that utilize the full scope of practice of non-dental professionals in custodial settings to integrate oral health into routine care, in addition to improving access to oral healthcare services, oral hygiene practices, and healthy dietary habits [15]. Policy makers, researchers and people with lived experience of incarceration can also play a role in the development of comprehensive oral health promotion strategies [16].

Oral healthcare remains one of the most neglected aspects of health in prison settings, particularly in terms of research, education, and service provision [6]. Few studies have attempted to address oral health in prisons. Published in 2023, Booth and colleagues’ scoping review documented three types of oral health interventions available in prison settings, which include health education, teledentistry, and screening/triaging [17]. Another review published by Evensen and colleagues in 2023 revealed the complex nature of oral health in prisons and the need for understanding its individual and organizational aspects through further research and investigation [18]. Despite previous efforts, various dimensions—such as prevalence, contributing factors, and interventions to improve oral health among incarcerated individuals—remain underexplored. To narrow this gap, the present qualitative study was designed to investigate the barriers and facilitators associated with providing and utilizing oral health services in prison settings, drawing on insights from prison health experts, managerial and custodial staff, healthcare providers, and individuals with lived experience of imprisonment.

## Methods

This qualitative study aimed to investigate the experiences of stakeholders regarding oral health in prison settings. Semi-structured in-depth interviews were conducted until data saturation to identify barriers and facilitators of providing and utilizing oral health services in prisons and explore potential areas for improvement. This approach aimed to understand the participants’ experiences, perceptions, and interpretations in their roles, capturing the richness and depth of their perspectives [16].

The primary framework utilized in this study is the WHO Prison Health Framework, which articulates the essential elements for equitable and efficient service delivery impacting oral health outcomes in prison populations [19]. This framework, which aims to assess prison oral health system performance, is based on the previous WHO frameworks, including the Health System Framework [20] and the Framework for Monitoring National Health Strategies [19]. Its development involved multiple meetings with the Health in Prisons Program (HIPP) Steering Group and the Health in Prisons European Database (HIPED) Technical Expert Group, comprising prison health experts [21].

### Ethical considerations

This study, following international guidelines on ethics in health research, was approved by the ethics committee of the Faculty of Medicine of Heidelberg University (approval number: S-288/2023). Data collection commenced after each respondent signed an electronic consent (e-consent) form including the clarification of the voluntary nature of participation, and their right to withdraw at any time. Interviews were conducted online utilizing Zoom, a video conferencing platform. To ensure participant confidentiality, no personal information was collected during the interviews. Any potentially identifying information was removed during the transcription and coding processes. Data were recorded digitally with anonymized file names, and audio files were deleted after transcribing.

### Participants and sampling

From March to June 2023 a purposive sampling approach was used to select key informant participants for this study. The research team sourced invitees’ contact details through professional networks and published papers of corresponding authors specializing in prison oral health. Written email invitations were then sent to selected experts based on their expertise and experience in prison oral health. After accepting the invitation, participants received detailed information about the study’s objectives, methods, and informed consent process. A total of 56 invitations were sent to experts in prison oral health, policymakers, and medical staff, including current and former prison doctors and dentists, from North America, Europe, and South America. A total of 15 (27%) of the 56 people who were contacted agreed to participate.

The experts interviewed included eight males and seven females, representing a diverse range of professional backgrounds. Among them were four prison dentists (DEN), one of whom also served as a regional dental director (DEN/RDD), and another as an oral health researcher (DEN/OHR). The group also included three prison doctors (DOC): a prison medical service manager (DOC/MSM), a medical administration member (DOC/MAM), and an infectious disease specialist (DOC/IDE). Additionally, two directors of prison education centres (DEC), one prison health policymaker (PHP), and two prison directors (DIR)—one of whom was also a nurse (NUR/DIR)—participated. The sample also included three prison health researchers (PHR). Notably, two participants had lived experience in prison (LEP).

Out of the 56 invited participants, 41 declined to participate. The group of non-respondents included 16 paper authors, 6 policymakers, 5 dentists currently working in prisons, 4 directors of prison foundations, 3 dental university professors, 2 group of dental students on internships in prisons, 2 correctional officers, 1 prison director, 1 nurse working in a prison, and 1 correctional law enforcement officer.

### Procedure

Each on-line Zoom interview ran for approximately 40 min and used an open-ended, interactive format to engage participants in discussions. Interviews were conducted by AA in English, and the participants were not remunerated. The interviews were recorded by a voice recorder, and recorded voices were deleted after being transcribed by AA, IM, and BM.

### Interview guide

An interview guide was designed by AA and BM and subsequently reviewed and finalized by the research team (Appendix 1). The interview guide consisted of seven main sections. The first section focused on the interviewee’s current positions and their connection to oral health in prisons. The second section explored the challenges of providing oral healthcare services in these settings. The third section gathered information about the level of access to oral health services in the prisons where the interviewee works. The fourth section addressed the barriers to providing and utilizing oral health services in prisons. The fifth section examined the facilitators that support the provision and use of oral health services. The sixth section inquired about existing policies and guidelines on oral health in prisons and the extent of adherence to these guidelines. Lastly, the seventh section explored the impact of the COVID-19 pandemic on the delivery of oral health services in prison settings. A pilot study with a prison health professional was conducted to ensure the face validity of the questions prior to the main interviews. Questions were modified for clinicians, non-clinicians, and policymakers based on their roles and expertise, guided by the WHO Prisons Health Framework [19].

### Data analysis

Thematic analysis was used to analyze the data, following Braun and Clarke’s method [22]. This interpretative approach facilitates identifying patterns or themes within the data. Themes and sub-themes were initially derived from the dataset and converted into preliminary codes aligned with research objectives. The analysis remained flexible, allowing for unexpected themes to emerge. MAXQDA 2022 software supported coding and condensing the data. Inductive codes were identified and organized into themes based on commonalities. The triangulation process integrated data from different interviews and compared findings with existing literature.

## Results

This section comprises barriers and facilitators of providing and utilizing oral health services in prison settings.

## Barriers

The first topic, *Barriers*, provides a key insight into the challenges of oral health service provision and utilization in prisons, experienced by PLP and custodial staff. The two themes that emerged from the interviews comprise: organizational barriers related to the provision of services (inclusive of five sub-themes), and individual barriers related to the utilization of services.

### Organizational barriers

The organizational barriers are presented under the following five sub-themes: lack of services; lack of knowledge and information; absence of prevention campaigns; lack of proper policies, and lack of preparedness during crisis.

#### Lack of services

The first and one of the most frequently discussed barriers mentioned in the interviews was the lack of oral health services in prison settings. The participants believed that in most countries, worldwide, oral health services are either unavailable or, if available, are offered with low accessibility, coverage, and quality. In some prisons that offer services, PLP are required to wait a long time to see a dentist and receive services. The interviewees mentioned four possible reasons associated with this issue:

- Lack of priority

The interviewees believe that most attention is given to the diagnosis, treatment, care, and support of infectious diseases such as HIV/AIDS, viral hepatitis, and tuberculosis, leaving oral health as a neglected issue in prison settings. One of the participants stated:

*“… prison officers don’t see it (oral health) as a priority. Not all of them. There are some who are good*,* and they understand the importance*,* but a lot of prison officers don’t think to get them. What they have at the moment is more than enough. And anyone who wants to improve education or information*,* especially about health care*,* it is not a priority*,* not at all.” *(DEC/LEP, Male, Europe)

- *Lack of resources*

The lack of sufficient resources, particularly budget and human resources, is another barrier to the availability, accessibility, and coverage of oral health services in prison settings, as underlined by the participants. One of the interviewees highlighted this issue as follows:

*“…there is very little resources for providing health intervention in prison. You know*,* because there are few people not much received financial resources*,* but also because not many people want to go and work in prison*,* because they are a specific population and there is fear*,* which is I think more*,* how do you say… dangerous.” *(DOC/MSM, Female, Europe)

Another participant pointed out the challenge of staffing prison settings:

*“We don’t have a dentist always at the institution because they make good money on private and then it is hard to find a dentist to work in prison settings.” *(DOC/MAM, Male, Europe)

- Reliance on tooth extraction

According to the participants, the availability of dental extractions as a cheap and rapid treatment option may influence treatment decisions in resource-limited settings such as prisons. The reliance on dental extractions as the standard treatment option may convince prison policymakers that more expensive services are not required in prison settings, as the interviewees mentioned. One of the participants highlighted this issue as follows:

“They’ll only do the bare minimum, if you’re in pain. They’ll extract it. That’s it.” (DEC/LEP, Male, Europe)

#### Lack of knowledge and information

The lack of information was another barrier to providing quality oral health services in prison settings, as discussed by the interviewees. The participants mentioned the absence of two levels of information. First, there was a lack of detailed information about the status of oral health among PLP collected by prison healthcare staff members. Second, there was a paucity of research-based information about how to design, implement, and monitor oral health interventions. One of the participants highlighted this issue as follows:

*“…better understanding of oral health needs, that it does require access in correctional facilities, particularly prisons, but also jails, which is where people are held for shorter periods of time, usually, for less serious crimes or awaiting trial. There’s really, really very limited research on this area….” *(DEC, Male, Europe)

In addition to the issues mentioned above, a lack of information regarding how to access and utilize services within the prison system represents another structural barrier that hinders PLP from accessing oral health care. As one participant noted:

*“…I would say there’s probably minimal information given to prisoners. It’s just, we have a dentist, or here you can apply to see the dentist. That’s it. The services that they offer you will find out when you get to see the dentist, but even then, it might be too late, or you might have done something yourself….”* (DEC/LEP, Male, Europe)

#### Absence of prevention campaigns

Prevention campaigns can help to raise awareness among the target population about diseases and inform them about evidence-based preventive activities, as discussed by the interviewees. The lack of such campaigns was discussed as another organizational barrier, as highlighted by one of the participants:

*“… and the only things is not only to ensure the presence of a dentist service but these dentists service is just doing these removing teeth… the point is to give prevention measures*,* sometimes at least once a year*,* a couple of times a year to make prevention campaign and they know they are not doing this…*.” (DOC, Male, Europe)

#### Inconsistent policies

According to the interviewees, the presence of clear and consistent policies is essential for the effective implementation of healthcare interventions across all sectors, including the prison system. In countries with federal political systems, regional policies may conflict with one another. Additionally, existing policies might be restrictive, such as those limiting the provision of certain products like dental floss to incarcerated individuals. As one participant noted:

“*One of the kind of challenges is that each state has their own procedures. And so it’s hard to put an extensive statement on what does access to root canals or something look like in prisons in the [name of the country redacted]*,* because likely looks very different in [name of the cities redacted] versus [name of the city redacted].” *(PHR, Male, North America)

#### Lack of preparedness during crisis

The participants believe that most prisons, worldwide, are not adequately prepared to function adequately during times of crises. In most prisons, the primary focus during the pandemic was on controlling the spread of the disease, thus reducing the availability of funding for human resources for other sectors, including oral health services. One of the interviewees highlighted this issue:

*“I don´t tell data but for sure I can tell you that there was a decline in the number of people in the dentist care, but on the paper all the specialist activities have been confirmed.”* (DOC/IDE, Male, Europe)

### Individual barriers

The second set of barriers discussed by participants were the individual barriers faced by PLP towards accessing and utilizing oral health services. The themes emerged from this topic include mistrust; pre-imprisonment neglect of oral health; stigma; and language barrier.

#### Mistrust

From the participant’s perspective, mistrust in the prison system, healthcare services, and healthcare providers is a significant factor contributing to the underutilization of services among incarcerated individuals. Many PLP do not trust the system that has deprived them of their liberties. Additionally, there is often a cultural issue of distrust towards female healthcare providers (e.g., physicians, dentists, and nurses) among male PLP, as the interviewees discussed. One participant highlighted this issue, stating:

*“…when I take a tooth out, they ask did you take it all? Because they don’t trust a woman can do this job….”* (DEN, Female, Europe)

#### Pre-imprisonment neglect of oral health

The interviewees believe that incarcerated individuals may have neglected their health, particularly oral health, even before imprisonment. This issue may be linked to a low perception of risk or severity of oral health conditions among PLP; this can also be associated with the lack of affordability of oral health services for lower socioeconomic groups of the community, where the majority of PLP come from. In this regard one of the participants stated:

*“My perception is that oral health is important only for the layers of population that can afford it.” *(DOC, Male, Europe)

#### Intersecting vulnerabilities

The participants believe that specific groups of PLP, including youths and older persons, are at an increased risk of poor oral health. Young people may neglect their oral health due to low perceived risks, while older people may already have poor oral health due to long-standing risk factors, as the participants discussed. This issue highlights the importance of focusing on at-risk populations, such as youths and older PLP, when providing and accessing oral health services in prison settings:

*“…we accredited dentists coming to prison, we also regulated prosthetic dental care for vulnerable prisoners. If these steps had been approved earlier, more of them could have benefited from better care….” *(DOC, Female, Europe)

#### Stigma

Stigma and the fear of discrimination may deter incarcerated individuals from seeking and utilizing oral health services, as they might wish to avoid receiving standard care. These issues are complex and require a comprehensive approach, as recommended by the interviewees. Such an approach should cover a variety of activities, including education for both PLP and healthcare providers. In this regard, one participant noted:

*“A lot of them [PLP] don’t have nice teeth, because they come from [name of the region redacted], and don’t have this level of health provision as the [name of the continent redacted], so they need education.”* (DEN, Female, Europe)

#### Language barrier

According to participants, for migrant and foreign national PLP, language barriers can be one of the main and most significant barriers affecting access and utilization of healthcare services. Due to the impaired ability to communicate, key oral health promotion messages including the availability and accessibility of oral health services in prisons may not have been received. In relation to access, PLP may experience challenges in communicating their oral health concerns to healthcare providers and receive appropriate care, as the respondents suggested. This issue is of great importance given many prisons lack financial support to provide access to interpreters:

*“…we have people from other countries*,* I want to explain that a tooth can’t be saved*,* it needs* *to be extracted*,* but they don’t always understand*,* or maybe they believe it can be fixed. I* *don’t always know their culture*,* and they don’t always trust what I’m saying.” *(DEN, Female, Europe)

## Facilitating factors

The second topic, facilitators, identifies elements that enable and enhance access to oral health services in correctional facilities through supportive measures for improvement and utilization. These factors are organized into the following six themes: funding, community partnership, substance use treatment, transportation, communication, and education.

### Funding

Securing funding is the first and one of the most important prerequisites for any intervention. In the context of oral healthcare delivery, funding is not only used for employing healthcare professionals but also for procuring products such as prosthesis (denture) glue, mouthwash, and floss. Ensuring sufficient funding for oral health services in prisons will guarantee the sustainability and quality of these services. One of the participants believes that:

*“…Resources are a major issue. Our national health service is government-funded through taxes but operates on very small budgets…” *(Prison Education Manager, Male, Europe)

### Community partnership

Partnership and collaboration with international and civil society organizations, including non-governmental organizations (NGOs), emerged as another factor facilitating access to oral health services in prison settings. Civil society organizations can help improve the quality of oral health services by securing external funding through advocacy and providing external healthcare providers in often understaffed and under-resourced settings such as prisons.

*“…We collaborate with the [name of the organization redacted], on various educational initiatives, such as promoting vaccine uptake and personal hygiene through poster campaigns and information days. A big push on a domain around mental health, but dental and oral health haven’t heard about it, which is quite telling…”* (DEC/LEP, Male, Europe)

### Substance use treatment

Substance use is common among the prison population. In addition to the mental and societal consequences, substance use can severely impact physical health, including oral health. The deterioration of oral health due to substance use can be direct or indirect. Substances can directly cause tooth decay, cavities, and bruxism. Additionally, substance use can lead to lifestyle changes that result in less attention to oral health and hygiene. In this context, treating substance use disorders can help improve the oral health of PLP who use drugs:

*“…The main issue with opioids and other substances in prisons is mental health. Inmates worldwide face significant mental health challenges, worsened by addiction to newer drugs like methamphetamine, leading to severe crisis and psychosis….”* (DOC, Male, Europe)

### Transportation

The interviewees believe that the availability of oral health units and providers in prisons offers the opportunity to address the urgent needs of PLP in a timely manner. However, providing such services is not always possible due to a lack of resources and other issues discussed earlier. To address this problem, participants recommend facilitating transportation from prisons to specialized services outside.

*“I understand that prison systems can’t always afford expensive dental units*,* but they [prison policy makers] can at least arrange transportation from the prison to community dental services. That’s indeed a far more affordable option.” *(PHR, Male, Europe)

### Communication

Participants identified communication with decision makers as a key facilitator that could enable the provision of oral health services within their prisons. While oral health can play a crucial role in the reintegration of PLP after return to the community, this can be communicated with prison policy makers to highlight the importance of providing quality oral health in prisons, as the respondents suggested. One of the interviewees stated that:

*“A lot of people leave prison and jail for oral health from a cosmetic standpoint*,* and that can become a huge barrier as they try to reintegrate into society. And they try to get a job that can be a barrier to get hired and when they decide to reform relationships and romantic partnerships.” *(PHR, Male, North America)

### Education

Peer education has been shown to be effective in increasing healthcare service utilization both in prisons and in the general population. Participants emphasized the importance of focusing on peer education as a method for delivering information to PLP and improving their oral health. Peer education can help overcome mistrust and skepticism toward health education programs, as recommended by the interviewees:

*“[providing] Education [in prison] is important. Everyone knows that. But even more important is who will deliver the education in prisons. In-prison peer education is an option. This [education] can also be done by externals like NGOs working with PLP. Doors must be open for those externals who want to enter into prisons and educate [PLP].”* (PHR, Male, Europe)

## Discussion

The present qualitative study identified organizational and individual barriers and facilitators of providing and utilizing oral health interventions in prison settings. From the participants’ perspective, two primary categories of barriers—organizational and individual—impede both the provision of and access to oral health services within prison settings. In addition, the interviewees identified several facilitating factors that could support the improvement of oral health among incarcerated individuals, which include funding, community partnership, substance use treatment, transportation, communication, and education.

The lack of availability, accessibility, coverage, and quality of healthcare services is a widespread issue in prisons [21]. This issue was also frequently discussed by the participants as an organizational barrier to improving oral health in prison settings. At the national level, lack of availability often stems from insufficient funding allocated to prison healthcare. At the institutional level, however, it may result from a prevailing misconception among policymakers, who view incarcerated individuals primarily as criminals to be punished rather than as individuals entitled to healthcare. While ensuring adequate budgets for prison healthcare is essential, alternative strategies can also be employed to address the shortage of healthcare professionals in these settings.

Lack of resources, in specific human resources, was another major barrier highlighted by the participants. The WHO Global Oral Health Strategy recommends interprofessional approaches to oral health promotion, utilising the full scope of different non-dental professionals [14]. In the custodial context, with further training, this interprofessional approach could involve correctional officers and healthcare professionals. This investment in training could lead to improved oral health through screening, education, treatment, and referral practices. One such example exists in the US, where the initial oral health assessment is conducted by trained correctional officers who then refer to a registered nurse (RN) for an intake evaluation [23]. The RN then refers to a physician or nurse practitioner for a full dental assessment and treatment plan within 14 days of reception, which then initiates a referral to the dental team.

To integrate oral health into routine custodial care, flexible, innovative workforce models of non-dental professionals are recommended to action the WHO Global Oral Health Strategy [14]. Integrated models of care have been demonstrated to be cost-effective, sustainable and effective in improving patient outcomes in non-custodial health settings such as cardiovascular health, midwifery, drug and alcohol, and diabetes which could be adapted to custodial settings [24]. Consequently, effective implementation of task shifting depends on several key factors, including collaboration among stakeholders, a coordinated care system, provider empowerment, patient preference, shared decision-making, comprehensive training and competency building, a supportive organizational framework, clear outcome processes, and adequate financing [25].

The lack of budget was another organizational barrier discussed by the interviewees of this study. To address this issue, mandatory health insurance and the separation of healthcare budgets from prison budgets—both at the institutional and central administration levels—are recommended as essential measures to ensure the affordability of healthcare services, including oral healthcare, within prisons [26]. However, in countries where people in prison are excluded from community health insurance programs, they experience higher rates of discontinuity in care and worse health outcomes after release from prison [27]. Moreover, evidence shows that the expansion of national insurance programs is associated with an increased likelihood of public health insurance coverage for formerly incarcerated individuals [28]. Therefore, health insurance coverage can play a crucial role in improving access to oral health services for the prison population, both during incarceration and after return into the community.

Distrust in prison healthcare system is the next barrier emerged from the discussions, which is a chronic and well established issue in prison systems [29]. The issue of distrust in prison healthcare system is multifaceted and may be associated with a wide range of factors. In our study, distrust in female dentists was reported to be an individual barrier towards providing and utilizing oral health services in prisons. While no studies have specifically evaluated the relationship between a physician’s gender and patient satisfaction within prisons, evidence from non-prison settings suggests that male patients treated by female physicians report significantly lower satisfaction levels compared to those treated by male physicians [30], which may be attributed to a lack of trust in female physicians. Further studies are needed to examine trust-related issues involving female healthcare providers, including dentists, in prison settings. Such research would provide the foundation for evidence-based recommendations to address and mitigate this concern.

Oral health is closely linked to personal behaviors including maintaining effective tooth brushing and flossing habits, attending regular dental check-ups, adopting dietary modifications such as reducing sugar intake, and minimizing risk factors like smoking and alcohol consumption [31]. Given this fact, education can play a crucial role in improving the healthcare status of individuals both in prisons and in the community, which was also highlighted by the participants as a facilitating factor. However, providing education to incarcerated individuals presents several challenges. For example, while peer education has proven effective in increasing the acceptability of educational programs in prisons, some countries prohibit individuals with a history of imprisonment from entering prisons, even to provide peer education [29]. This restriction eliminates peer education as an option in these contexts and jeopardizes the impact of educational programs in prisons. Given the complex nature of prison environments and the unique needs of incarcerated individuals, prison systems must explore every avenue to provide quality oral health services, including incorporating peer education programs.

Oral health in custodial settings is a complex and multifaceted issue. Therefore, addressing oral health in prisons requires a comprehensive and multidimensional approach [31]. McNeil et al. suggest four key areas in oral health which comprise “behavioral and social theories and mechanisms related to oral health, use of multiple and novel methodologies in social and behavioral research and practice related to oral health, development and testing of behavioral and social interventions to promote oral health, and dissemination and implementation research for oral health” [32]. While addressing these areas is essential for delivering quality oral health interventions in prisons, it is crucial to ensure that all such interventions are specifically tailored to the unique needs of incarcerated individuals to be effective in this complex setting.

## Limitations

Our study had some limitations. First, the number of participants with a history of imprisonment in the interviews was limited. Second, most interviewees were from high-income countries, which may have restricted insights into oral health in prisons within low- and middle-income settings. Third, the limited number of female participants reduced our ability to explore oral healthcare among the female prison population. Fourth, the inclusion of participants from three different world regions may have influenced the comparability of their experiences—particularly between policymakers and medically trained staff—as they were grouped together for analysis and data saturation purposes. Finally, due to challenges in accessing individuals currently living in prisons and conducting research in such settings, we were unable to interview this group, which may affect the scope of our findings. Addressing these limitations in future research will lead to more reliable and generalizable information.

## Conclusions

This study identified several barriers and facilitators in providing and utilizing oral health services from the perspectives of prison health policymakers, healthcare providers, researchers, and individuals with a history of imprisonment. These findings should inform the design and implementation of oral health services in prison settings. Ensuring the oral health of incarcerated individuals is not only a fundamental aspect of their right to health but also a crucial factor in their successful reintegration into society upon release. Imprisoned individuals are often hard to reach when in the community, making incarceration a unique opportunity to deliver quality oral healthcare. Given that most incarcerated individuals will eventually return to their communities, providing comprehensive healthcare, including oral health interventions, represents a valuable public health investment.

## Supplementary information

Below is the link to the electronic supplementary material.

ESM 1 (PDF 127 KB)

## Data Availability

The study materials, including the transcripts of the discussions, are available to share with the editorial and review team upon request Arianna Amaya (arianna.amaya@uni-heidelberg.de) should be contacted to obtain access to the raw data analyzed in this study.

## Abbreviations

DEC: Director of Medical Administration in Prison
DEN: Prison Dentist
DIR: Prison Director
DOC: Prison Doctor
COVID-19: Coronavirus disease 2019
HIPED: Health in Prisons European Database
HIPP: Health in Prisons Program
HIV/AIDS: Human Immunodeficiency Virus and AIDS Acquired Immunodeficiency Syndrome
LEP: Lived Experience in Prison
MAM: Medical Administration Member
MSM: Prison Medical Service Manager
NGOs: Non-governmental organizations
NUR: Nurse
OHR: Oral Health Researcher
PHR: Prison Health Researcher
PLP: People living in prisons
RN: Registered Nurse
SDGs: Sustainable Development Goals
UHC: Universal Health Coverage
WHO: World Health Organization
