# Supplementary material for: Understanding the complexities of oral healthcare delivery in correctional settings: a qualitative exploration of barriers, facilitators, and opportunities
Source: BMC Public Health. 2025 Sep 9;25:3039. doi: 10.1186/s12889-025-24447-9 (PMC12418666; doi:10.1186/s12889-025-24447-9)
Supplement: Supplementary file 1 — (PDF 127 KB) [file 12889_2025_24447_MOESM1_ESM.pdf]

**Appendix 1. Semi-structured in-depth interview guide**

Oral health services in prison settings: A comprehensive assessment of availability, accessibility and model of delivery

**I. Introduction and Consent**

Thank you for agreeing to participate in the study about oral health in prisons. I am Arianna Amaya, a master's student at the Heidelberg Institute of Global Health. Your cooperation and availability to take part in this interview is valuable input to this study. I am conducting a study on the assessment of the availability, accessibility, and model of delivery of oral health services in prison settings. To capture all the details of the interview, I would like to ask your permission to use a recorder. Your personal information will remain confidential. After the interview, I will transcribe and then delete the recording. If you feel there is something you would like to share but would not want to be recorded, please let me know and I will pause the recording to do so. If I have your consent to start the interview, I would kindly like to start it now. If you have any questions now, I am happy to answer them.

Thank you for coming. Please let us get started.

**I. Interview guide**

| **Main Questions** | **Follow up Questions/points** | **Probes** |
|---|---|---|
| How would you describe your influence on oral health provision in the custodial setting? | Region, prison setting? | Aha… |
| As a dentist/expert what is needed in prisons to achieve oral health? | How can be your work done better? | Nodding… |
| How do you perceive the level of access to dental services among PLP in the facility where you work or region? | Are there any specific barriers or challenges that impact their access to care? | I see… |
| Can you describe any notable challenges you have faced or aware of while providing dental care to PLP? | How have you addressed them? | I get that… |
| How is (do you) assess the oral health status of PLP in | (Demonstrating involvement by non-verbal means) | How is (do you) assess the oral health status of PLP in |

| | | |
|---|---|---|
| your region, and what indicators are used to monitor and evaluate their progress over time? | | your region, and what indicators are used to monitor and evaluate their progress over time? |
| What would you like to apply in this setting or region? | Ok… | What would you like to apply in this setting or region? |
| In your experience, what factors have facilitated the provision of high-quality dental care to prisoners? | Clear | In your experience, what factors have facilitated the provision of high-quality dental care to PLP? |
| How do you view your role as a dentist/prison health/expert working in a prison setting regarding oral health improvement? | How balanced are the dual responsibilities of providing clinical care and promoting oral health education? | |
| How are dental services in your region/prison aligned with the prison guidelines and protocols? | | |
| How did the COVID-19 pandemic change the oral health services in your region? | Are those changes still being implemented? | |

I believe we are nearing the end of our discussion. Allow me to summarize the key points we have covered today. It appears that you hold the following beliefs or opinions based on our conversation… (summary of the key points and confirmation of the answers that the interviewer would like to clarify). I sincerely appreciate the time and effort you have dedicated to this interview; your input is extremely valuable for our study. If we require further clarification or follow-up in the future, would it be possible to reach out to you for a brief additional meeting?

Once again, thank you for your participation. I hope you have a nice day.
